# The analysis of schizophrenia-like psychosis in dentatorubral-pallidoluysian atrophy

**DOI:** 10.3389/fneur.2025.1564856

**Published:** 2025-04-09

**Authors:** Ichiko Ikegami, Yuka Mitsuhashi Koike, Hideki Hayashi, Sachiko Hirokawa, Shoichiro Ando, Tomohiko Ishihara, Osamu Onodera

**Affiliations:** ^1^Department of Neurology, Brain Research Institute, Niigata University, Niigata, Japan; ^2^Department of Molecular Neuroscience, Brain Research Institute, Niigata University, Niigata, Japan; ^3^Advanced Treatment of Neurological Diseases Branch, Brain Research Institute, Niigata University, Niigata, Japan

**Keywords:** dentatorubral-pallidoluysian atrophy, polyglutamine disease, psychosis, schizophrenia, psychotropic medications

## Abstract

**Background:**

Dentatorubral-pallidoluysian atrophy (DRPLA) is a progressive neurodegenerative disorder caused by expanded CAG repeats in the *ATN1* gene, characterized by cerebellar ataxia, seizures, tremors, and myoclonus. Although approximately 10% of patients with DRPLA reportedly develop schizophrenia-like psychosis (SLP), the distinct association between the clinical course of DRPLA and SLP remains unclear. This study aimed to elucidate the clinical features of SLP in patients with DRPLA.

**Methods:**

We reviewed 22 cases of pathologically or genetically confirmed DRPLA with SLP, including 21 from the literature and one from our institution. Patient data, including clinical features, treatment information, and disease course, were extracted and analyzed.

**Results:**

The age of onset was categorized as juvenile (*n* = 6), early adult (*n* = 8), and late adult (*n* = 8). Initially, 10 patients presented with motor symptoms, with six exhibiting psychiatric symptoms and six with both motor and psychiatric symptoms simultaneously. Furthermore, three patients were initially diagnosed with schizophrenia, while four experienced progressive worsening of psychiatric symptoms. The number of CAG repeats ranged from 57 to 76 (mean, 66.0) in the 10 patients with a genetic diagnosis. Summarily, 12 patients received psychotropic medications, with nine showing improvement in delusions and hallucinations.

**Conclusion:**

SLP can manifest across all DRPLA forms (juvenile-, early adult-, and late adult-onset) and may precede or follow motor symptoms. The clinical course and efficacy of psychotropic medications in patients with DRPLA and SLP suggest a shared pathogenesis between DRPLA and schizophrenia.

## 1 Introduction

Dentatorubral-pallidoluysian atrophy (DRPLA) is a progressive neurodegenerative disorder caused by expanded CAG repeats in the *ATN1* gene (1, 2). The age of onset and the severity of clinical manifestations correlate closely with the number of CAG repeats, with longer repeats typically leading to more severe disease at an earlier age (3). DRPLA occurs relatively frequently in Japan, with an incidence of 2–7 per million (4, 5), but remains rare in Europe and North America (6).

The clinical onset of DRPLA ranges from infancy to adulthood. Patients whose symptoms begin before the age of 20 years (juvenile-onset) commonly experience myoclonus, epilepsy, ataxia, and progressive cognitive decline. In contrast, individuals whose disease onset occurs at 40 years or older (late-onset) often present with cerebellar ataxia, athetosis, and dementia (7). Psychiatric manifestations, such as depression, anxiety disorders, personality alterations, and mood lability, have been documented throughout the disease course (7). Approximately 10% of patients with DRPLA reportedly develop schizophrenia-like psychosis (SLP) (8), which may include delusions and hallucinations. Importantly, psychotic features sometimes precede prominent motor dysfunction, leading to diagnostic uncertainty and possible misdiagnosis of primary schizophrenia (8, 9). Although SLP has been reported in adolescent-onset patients with mild intellectual impairment (9), the precise relationship between specific DRPLA subtypes and psychiatric symptoms remains unclear. Thus, in this study, we aimed to elucidate the clinical features and course of SLP in patients with DRPLA.

## 2 Methods

### 2.1 Literature search

We conducted a literature review to identify cases of DRPLA presenting with symptoms of SLP. In this study, SLP was defined as psychosis with distinct delusions and hallucinations. Other psychiatric symptoms, such as emotional numbing and thought disorder, were excluded due to the difficulties in identifying a clear onset and progression. A comprehensive literature search was performed in multiple electronic databases, including PubMed/MEDLINE and Japanese databases (Medical Journal Search/Ichushi Articles), from their inception to August 1, 2024. Given the significant number of relevant case reports originating from Japan, we emphasized thoroughly searching for Japanese-language sources. The search strategy employed a combination of Medical Subject Headings (MeSH) terms and free-text terms. For English databases, we used the terms “DRPLA,” “dentatorubral-pallidoluysian atrophy,” “Naito-Oyanagi disease,” “Haw River syndrome,” or “myoclonic epilepsy with choreoathetosis” in combination with “psychiatric,” “psychosis,” “schizophrenia,” “delusion,” “hallucination,” “mental,” or “behavioral.” Equivalent terms were used for the Japanese database searches. The references of the identified articles were manually searched for other relevant studies.

### 2.2 Case selection

Studies were eligible for inclusion if they reported on patients with either genetically confirmed DRPLA (documented CAG repeat expansion), pathologically confirmed DRPLA at autopsy, or cases with a clear family history for DRPLA and included descriptions of SLP symptoms, particularly delusions and hallucinations or specific episodes suggestive of such symptoms. We considered original articles, case reports, and case series published in English or Japanese with full texts available. We reviewed 27 reports in the initial search and excluded 13 reports for the following reasons:

- Absence of detailed description of psychiatric symptoms.
- Lack of pathological or genetic confirmation of DRPLA.
- Duplicate reports from the same patient.
- Studies focusing exclusively on motor symptoms without a psychiatric description.

Two reviewers independently screened the titles and abstracts of the identified articles, and disagreements were resolved by consensus or consultation with a third reviewer. In addition, through a full-text review to select literature cases, we confirmed that the genetic and/or pathological diagnosis of DRPLA was firm and that there was a clear description of delusions and hallucinations. We subsequently performed data extraction from the selected cases using standardized forms. The limitations of our review methodology, including the retrospective nature of the included studies and the potential publication bias, were explicitly considered in the analysis and interpretation of the findings.

Consequently, we identified 21 cases of SLP from 14 articles, including 20 cases of delusions and 17 cases of hallucinations (8–21). Additionally, we reviewed the records of 16 consecutive patients with DRPLA admitted to our institution’s Department of Neurology between 1985 and 2023, identifying one patient with prominent delusions and hallucinations. This identification yielded a final cohort of 22 patients with documented SLP. From each included case, we extracted data regarding patient profiles (age, sex, age at onset, and CAG repeat length, if available), clinical features (type and timing of psychiatric symptoms, neurological manifestations, and cognitive status), treatment information (medications used, response to treatment), and disease course. We classified cases based on the temporal relationship between motor and psychiatric symptoms into three categories: (1) psychiatric symptoms occurring at least 6 months after neurological onset, (2) psychiatric and neurological symptoms appearing within 6 months, or (3) psychiatric symptoms preceding neurological signs for at least 6 months. While providing quantitative summaries where appropriate, we adopted a narrative synthesis approach due to the predominance of case reports in the dataset.

## 3 Results

### 3.1 Patient profiles

Among the 22 selected patients (including one of our own 16 consecutive), 11 were male (50.0%), and 11 were female (50.0%). Regarding the age of onset, 6 were juvenile type (onset ≤20 years), 8 were early adult type (onset between 20 and 39 years), and 8 were late adult type (onset ≥40 years). Pathological confirmation was obtained for 11 individuals, while the remaining 11 were genetically diagnosed. CAG repeat lengths were identified for 10 genetically confirmed individuals.

### 3.2 Association between CAG repeat length and age of onset for SLP

Among the 10 genetically confirmed patients, the mean CAG repeat length was 66.0 (range 57–76). A Spearman correlation coefficient of 0.0800 (*p* = 0.8263) indicated no statistically significant relationship between the CAG repeat number and the interval from the initial DRPLA symptoms to the onset of delusions or hallucinations.

### 3.3 Neuropsychiatric symptom profiles

Ataxia was observed in 20 of 22 patients (90.9%), choreiform movements in 14 (63.6%), seizures in 15 (68.2%), and myoclonus in 7 (31.8%). In parallel with these movement abnormalities, a high proportion of patients, 20 of 22 (90.9%), exhibited some degree of cognitive decline consistent with dementia. Additionally, 18 patients (81.8%) presented with disinhibition, while 3 (13.6%) had a marked spontaneous reduction in social interaction. The psychiatric manifestations of SLP include agitation, paranoia, grandiose and persecutory delusions, and auditory and visual hallucinations. Examples of patients’ delusional content ranged from the belief that a person they had quarreled with was disseminating negative information via radio waves to statements such as “People around me are spying on me” or “I see a man in the corner of the room.” These manifestations closely resembled the hallucinations and delusions seen in schizophrenia.

### 3.4 Timing of psychiatric symptoms in relation to neurological presentation

**Figure 1:**
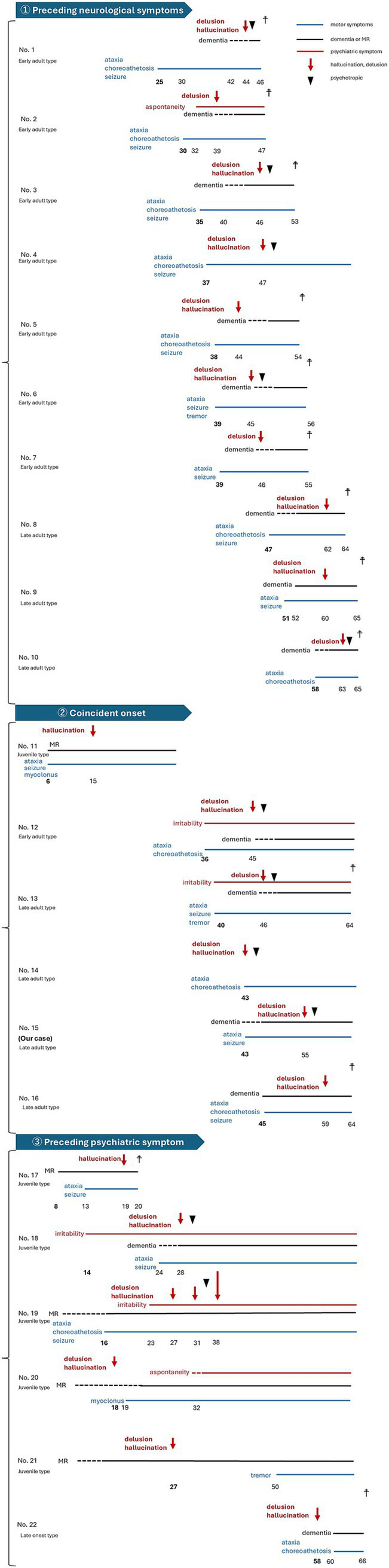
Clinical courses of 21 dentatorubral-pallidoluysian atrophy cases included in the analysis. The numbers in the figure indicate the onset of each symptom, and the numbers highlighted in bold indicate the age at which the first symptom developed.

Among the 22 patients, 10 developed prominent motor signs before psychiatric symptoms, 6 displayed psychiatric symptoms first, and 6 experienced a nearly simultaneous onset of motor and psychiatric disturbances (Figure 1). All ten patients presenting with motor symptoms before psychosis had early or late adult-type DRPLA, and the duration between the onset of motor symptoms and psychiatric symptoms averaged 9.7 years (range 6–19 years; median: 9 years). Conversely, among the six patients presenting with psychosis before motor symptoms, five were of the juvenile type, and the duration between the onset of psychiatric symptoms and motor symptoms averaged 7 years in the three cases with detailed records (range 2–10 years; median: 5 years). In 4 of the 22 cases (18.2%) with psychiatric symptoms, psychosis worsened dramatically, either following generalized seizures or triggered by physical discomfort. These acute exacerbations often present with hyperexcitability. Furthermore, in three of these four cases, rapid deterioration occurred after a seizure, with symptoms persisting without improvement.

### 3.5 Treatment response

Antipsychotic medications were prescribed to 12 of 22 patients (54.5%) of patients, with 9 (40.9%) reporting symptomatic improvement in delusions and hallucinations. The treatment agents included atypical (risperidone, olanzapine, and quetiapine) and typical (haloperidol, levomepromazine, and chlorpromazine) antipsychotics, sometimes in combination. Additionally, two patients (9.1%) described aggravations of extrapyramidal symptoms once antipsychotics were initiated. In one of the cases described (No. 8), the delusions spontaneously disappeared after a certain period, and no medication was used. Six patients, including three whose psychiatric symptoms seemed to worsen in the wake of seizures, also received anti-seizure medications, such as phenytoin, phenobarbital, valproic acid, carbamazepine, zonisamide, diazepam, or levetiracetam. Although some of these antiepileptic agents stabilize the epileptic components of DRPLA, none showed obvious benefits in alleviating psychotic symptoms.

### 3.6 Representative cases from our institution

#### 3.6.1 Case no. 15 (our case)

A 43-year-old female patient presented with executive dysfunction and gait disturbances; she had difficulty performing activities of daily living, such as locking doors and lighting stoves. Her mother had been diagnosed with DRPLA, and genetic testing confirmed her diagnosis (65 repeats). As the disease progressed, the patient developed symptoms suggestive of absence seizures, characterized by behavioral arrest and further cognitive decline. She was not receiving medication due to the side effects of anti-seizure treatments, leading to an escalation in seizure frequency, accompanied by a progressive decline in ambulatory function. At the age of 51, she was admitted to our hospital due to intermittent hallucinations and delusions, as well as worsening gait impairment. These symptoms were precipitated by abdominal discomfort, and she voiced statements such as, *“I will be buried in the ground”* and *“Rocks are chasing me.”* A thorough evaluation was performed, and the patient was admitted to the department with restlessness, cognitive decline, a dizzying change in language function and limb ataxia accompanied by hallucinations and delusions. An initial assessment of potential epileptic activity revealed the presence of alpha waves; however, no spasmodic activity was detected on electroencephalography (EEG) performed under sedation. Subsequently, a head MRI demonstrated diffuse brain atrophy involving the brainstem and cerebellum, with no signal changes in the cerebral white matter. Despite these findings, she struggled to maintain an upright posture, and her motor function deteriorated rapidly. Consequently, the patient was transferred to another medical facility for further evaluation and treatment.

## 4 Discussion

This study retrospectively analyzed the clinical course, prevalence, and spectrum of SLP symptoms in patients with DRPLA. In addition, the clinical course of a consecutive DRPLA case with schizophrenia-like psychiatric symptoms in our department was described in detail. The results emphasize the diverse presentation of psychosis in this disease, encompassing paranoia, grandiosity, auditory and visual hallucinations, and marked behavioral and cognitive changes. This frequency was observed in 1 of 16 consecutive cases, consistent with previous reports of approximately 10% (8). Notably, SLP has also been reported in polyglutamine disorders, including Huntington’s disease (22) and SCA17 (23, 24), with their frequency in Huntington’s disease ranging from 5 to 16% (25). These findings suggest that clinicians should maintain similar vigilance for developing SLPs in DRPLA as in Huntington’s disease.

Although previous studies have indicated that SLP may be particularly frequent in juvenile-onset DRPLA with mild intellectual disability (9), our findings indicate that such psychiatric manifestations can arise in any age-of-onset subtype. The appearance of psychosis and its relationship with motor onset vary. In some instances, SLP precedes discernible neurological deficits, sometimes for several years. In some cases, motor symptoms and cognitive or psychotic features emerge almost simultaneously, while in others, motor symptoms such as ataxia or chorea appear long before SLP. This variability suggests that multiple pathophysiological pathways underlie DRPLA’s neuropsychiatric expression. While our findings highlight that juvenile-onset patients were more likely to experience psychiatric symptoms before obvious motor manifestations, several adult-onset patients also developed SLP before the onset of characteristic DRPLA symptoms. These observations support the notion that similar to other polyglutamine expansion disorders, psychiatric disturbances may develop relatively independently of the severity or distribution of motor signs.

An additional point of interest is the rapid deterioration of psychiatric status in certain patients, particularly following generalized seizures or acute physiological stressors. These abrupt declines, often manifesting as hyperexcitability or prolonged psychosis, may reflect an amplified response in vulnerable neural circuits. Although postictal psychosis has been proposed as an explanation, the persistence of psychosis in some instances suggests that once destabilized, the cortical and subcortical networks supporting cognition and behavior may not fully recover. This observation is reminiscent of other neurodegenerative conditions in which acute metabolic or epileptic events can lead to long-lasting consequences on disease progression and quality of life.

Treatment responses highlight the complexity of the psychiatric symptoms in patients with DRPLA. Our analysis showed that nine of the 12 patients receiving antipsychotic medications experienced symptomatic improvement, while antipsychotics, both typical and atypical, produced improvement in more than one-third of the treated patients. However, there were also instances of exacerbated extrapyramidal symptoms, suggesting that, as in Huntington’s disease, clinicians must carefully balance the potential benefits of controlling delusions and hallucinations with the risk of worsening movement disorders, especially given patients’ preexisting basal ganglia pathology (25, 26). Furthermore, while many patients experiencing seizures receive anti-seizure medications, these agents offer little to no benefit for psychotic symptoms, indicating that seizure control, although important, may not translate into improved psychiatric outcomes.

From a neuropathological standpoint, several lines of evidence suggest that cortical inhibitory interneuron dysfunction plays a pivotal role in schizophrenia (27, 28). Particularly, parvalbumin-positive interneurons, which modulate local circuit oscillations and maintain excitatory-inhibitory homeostasis, are disrupted in many neuropsychiatric conditions (29). Studies have shown alterations in GABAergic interneurons in the prefrontal cortex (27, 30). Such disturbances in the GABAergic system may significantly contribute to psychiatric and cognitive symptoms. DRPLA autopsy studies have noted an extensive loss of both parvalbumin- and calbindin-D28K-positive interneurons in the neocortex (31), which may be similar to the alterations that occur in primary psychotic disorders such as schizophrenia. Further detailed studies, such as coupling imaging, electrophysiology, and postmortem tissue analyses, would be valuable for clarifying how synaptic and cellular network alterations translate into psychiatric phenotypes.

This study has several limitations. First, it is a retrospective study relying on variably published literature. Therefore, the longitudinal courses of symptoms are not followed and documented. Second, the divergent descriptions of psychosis and variable diagnostic thresholds from different sources may reduce the clarity of data comparison. Third, focusing primarily on Japanese sources may also limit the generalizability of our findings, given the genetic and environmental differences across global populations. Lastly, the relatively small number of cases with documented CAG repeats prevented us from establishing a more conclusive genotype–phenotype correlation regarding psychotic symptoms. Prospective research supported by international collaborations and uniform diagnostic criteria for both movement and psychiatric manifestations will enable a more precise understanding of the complexities of DRPLA-associated psychosis. Further studies would help to clarify any mechanistic links to specific CAG repeat lengths, providing fresh insights into how polyglutamine expansions disrupt the neural circuits that govern thought, mood, and perception.

In conclusion, our study demonstrates that SLP symptoms can develop in DRPLA regardless of the disease subtype or age of onset. These symptoms are not always secondary to motor dysfunction and may occasionally serve as early or initial presentations. Moreover, the sudden worsening of psychosis following seizures or physiological stressors indicates that the trajectory of psychiatric decline may be variable and catastrophic. These observations underline the importance of comprehensive monitoring of psychiatric features in patients with DRPLA and the need to optimize management strategies that account for a range of neurological and behavioral complications. Further investigations, particularly multicenter and international collaborations, are essential to deepen our understanding of the timing, progression, and pathophysiology of the psychiatric manifestations of DRPLA and related neurodegenerative conditions.

## Data Availability

The original contributions presented in the study are included in the article/Supplementary material, further inquiries can be directed to the corresponding author.
